# Acute changes in postural stability during Instagram Reels viewing using virtual reality–based posturography

**DOI:** 10.3389/fnbeh.2026.1808641

**Published:** 2026-04-29

**Authors:** Hanifi Korkmaz, Yasin Tok, Rania Alkahtani, Hadeel Alsaleh, Reem Elbeltagy

**Affiliations:** 1 Medical Health Services and Vocational School, Malatya Turgut Ozal University, Malatya, Türkiye; 2 Department of Child Development, Faculty of Health Sciences, Inonu University, Malatya, Türkiye; 3 Department of Health Communication Sciences, College of Health and Rehabilitation Sciences, Princess Nourah Bint Abdulrahman University, Riyadh, Saudi Arabia

**Keywords:** cognitive load, Instagram, postural control, REELS, short video addiction, stability, visual-cognitive abilities

## Abstract

Short-form video platforms such as Instagram Reels deliver continuous high–visual-flow stimulation that imposes substantial visual–cognitive load even in the absence of active motor interaction, yet their impact on postural control remains largely unexplored. This study examined whether passive Instagram Reels viewing acutely alters postural stability in healthy adolescents and young adults, and whether addiction severity and habitual use patterns modulate this effect. Regular Instagram users aged 16–21 years completed virtual reality–based static posturography before and during passive Instagram Reels viewing (i.e., viewing short-form, algorithm-driven video content without active user interaction such as scrolling or screen contact). Instagram addiction severity and usage habits were assessed, and their associations with viewing-related postural changes were evaluated. Vestibular-related postural stability decreased during Reels viewing. Higher Instagram addiction severity and longer daily Instagram use were associated with smaller reductions in vestibular-related postural stability between pre- and during-viewing conditions, a pattern compatible with partial habituation and/or compensatory postural control strategies. The purpose of Instagram use was not meaningfully associated with the magnitude of change. Passive short-video exposure can acutely compromise vestibular-related postural stability even during quiet standing, while habitual exposure appears to attenuate the immediate destabilizing effect. These findings extend smartphone–balance research toward a more ecologically relevant exposure model and provide a reference framework for future studies in clinically vulnerable populations, future studies in clinically vulnerable populations, such as individuals with vestibular disorders, neurological conditions, or balance impairments.

## Introduction

Short video–based social media platforms, such as TikTok and Instagram Reels, have become a dominant form of digital engagement among adolescents and young adults. These platforms deliver rapid, continuous visual flow and salient stimuli that impose substantial visual–cognitive load, even in the absence of active motor interaction (Ozkul et al., 2020; Luo et al., 2025). Accumulating evidence indicates that intensive short-form video consumption is associated with impaired attentional processes and increased cognitive load (Yan et al., 2024; Mona et al., 2026), factors that are particularly relevant for concurrent motor performance. When such cognitively demanding visual exposure occurs during standing, it may interfere with the attentional and multisensory processes required for stable postural control.

In this context, understanding how cognitive and visual demands translate into balance disturbances requires consideration of the mechanisms underlying postural regulation. Postural control is a dynamic process that relies on the continuous integration of visual, vestibular, and somatosensory inputs, and in everyday life it is frequently executed alongside concurrent cognitive activities (Woollacott and Shumway-Cook, 2002; Horak, 2006). Accordingly, social media consumption, particularly via short-form video content characterized by rapid visual flow and salient stimuli, may influence balance control not only through mechanical loading but also via the allocation of cognitive resources and the processing of visual information. This experimental paradigm can be explicitly framed within the dual-task framework, in which postural control is performed concurrently with a visual–cognitive task. In such conditions, competition for limited attentional resources has been shown to impair postural stability and alter center-of-pressure dynamics (Woollacott and Shumway-Cook, 2002). More recent evidence further demonstrates that visually demanding tasks, including smartphone use and visual dual-task conditions, can modify postural control strategies and increase biomechanical and attentional load (Brühl et al., 2023; Chen and Nguyen, 2023; Ghamari et al., 2023). Accordingly, passive exposure to high–visual-flow short-form content may represent a distinct form of visual–cognitive dual-tasking that challenges multisensory balance regulation.

Empirical evidence supporting this theoretical framework has increasingly measurable balance alterations during smartphone use. A growing body of literature suggests that smartphone use can adversely affect postural stability in healthy individuals. Studies examining texting, social media interaction and selfie taking have consistently demonstrated increased postural sway and deterioration of center-of-pressure–based balance metrics accompanied by significant impairments in temporal and spatial postural metrics, particularly under challenging sensory conditions (Noll et al., 2024). Prolonged smartphone use has been linked to short-term decrements balance performance, with exposures of 20–40 min further amplifying this effect (Jouira et al., 2023). However, most existing studies remain limited in ecological validity, predominantly focusing on texting or controlled smartphone interactions, thereby insufficiently representing real-life behaviors involving rapid visual flow, such as short-video consumption (Noll et al., 2024). Moreover, the majority of available evidence derives from either laboratory-based paradigms or clinical populations, leaving uncertainty regarding how such digital exposures influence postural control in neurologically and musculoskeletally healthy individuals. In this framework, postural stability is conceptualized as a functional sensorimotor performance outcome modulated by attentional allocation rather than a purely peripheral balance function.

In addition to balance-focused findings, a growing body of research has highlighted the biomechanical and postural consequences of smartphone use. Experimental and observational studies have demonstrated that smartphone-related activities lead to increased cervical flexion, changes in spinal alignment, and altered biomechanical loading (Betsch et al., 2021; Brühl et al., 2023; Chen and Nguyen, 2023). Furthermore, prolonged smartphone use has been associated with postural deviations and functional changes, including respiratory alterations and increased musculoskeletal strain (Jung et al., 2016; Walankar et al., 2021). Together, these findings suggest that smartphone exposure may influence postural control not only through cognitive load but also via biomechanical and sensorimotor mechanisms.

This gap is particularly salient when age-related developmental factors are considered. Adolescence and early adulthood represent a particularly relevant developmental window for examining these effects. During this period, attentional control, sensory integration, and postural regulation systems continue to mature, while digital media use intensifies and increasingly occupies prolonged periods of daily activity. In parallel, device-related postural habits have been linked to musculoskeletal strain and altered movement strategies (Tsang et al., 2023). Beyond biomechanical factors, heightened reliance on visual input and divided attentional resources may render postural control more vulnerable during visually demanding digital exposure (Gong et al., 2018; Ivanenko and Gurfinkel, 2018).

Accordingly, objective and sensitive assessment tools are required to disentangle the specific components of postural control affected by digital exposure. Virtual reality (VR)–based static posturography enables controlled manipulation of sensory conditions and objective quantification of postural responses, allowing the estimation of the relative contributions of visual, vestibular, and somatosensory systems to balance control rather than their direct isolation (Horak, 2006; Rosiak et al., 2022).

In the present study, sensory contributions were differentiated using a VR-based Clinical Test of Sensory Interaction on Balance (CTSIB) framework in which somatosensory input was reduced via foam surface conditions, visual input was manipulated through eyes-open/closed and dynamic VR visual stimuli, and visual–vestibular conflict was induced using sway-referenced visual motion. This condition-specific manipulation allows indirect estimation of sensory weighting based on postural performance patterns (Horak, 2006; Meldrum et al., 2012). When combined with the Limits of Stability (LOS) testing, this approach provides a comprehensive framework for assessing both sensory integration and functional balance components and has been widely applied in balance research. Establishing baseline postural responses in healthy adolescents using such objective methods is a critical prerequisite for interpreting digital-exposure–related balance alterations and for contextualizing findings in clinically vulnerable populations.

Despite these methodological advances, important knowledge gaps remain. Studies that directly compare pre- and during-exposure postural performance in response to high–visual-flow short-video content within ecologically relevant paradigms remain scarce (Jouira et al., 2023; Noll et al., 2024) Moreover, little is known about whether habitual exposure and problematic use patterns modulate the magnitude of any acute postural destabilization.

Therefore, the present study aimed to investigate the immediate impact of passive Instagram Reels viewing on postural stability in regular Instagram users aged 16–21 years, with particular emphasis on vestibular-weighted components of postural control. A secondary aim was to examine whether Instagram addiction severity and habitual usage characteristics modulate the magnitude of viewing-related postural changes. By integrating high–visual-flow digital exposure into a VR-based postural assessment framework, this study sought to provide an ecologically grounded reference model for understanding digital media–related balance alterations and to inform future research in clinically vulnerable populations.

## Materials and methods

### Research design

This study adopted a within-subject, repeated-measures design integrated with a correlational analytical framework to examine both (i) the association between Instagram addiction severity and postural stability and (ii) the acute effects of Instagram Reels viewing on postural control. Each participant was assessed under two experimental conditions: a baseline measurement conducted prior to Reels exposure and a second measurement performed during passive Reels viewing. This design enabled direct within-individual comparisons of postural performance while minimizing inter-individual variability. The same postural assessment protocol (CTSIB-VR) was applied in both conditions (pre- and during-Reels viewing). Limits of Stability (LOS) testing was conducted only during the baseline assessment and was not performed during the Reels viewing condition due to its requirement for active voluntary weight shifting.

Postural stability was evaluated using virtual reality (VR)-based static posturography across multiple sensory conditions in conjunction with Limits of Stability (LOS) testing, providing a comprehensive assessment of multisensory integration and functional balance capacity. The primary within-subject factor was measurement time (pre-viewing vs. during-viewing), whereas Instagram addiction scores and habitual usage characteristics were treated as continuous explanatory variables in correlational and subgroup analyses.

The study protocol was approved by the Malatya Turgut Özal University Clinical Research Ethics Committee (Decision No: 2025/392; Date: October 16, 2025), and all procedures were conducted in accordance with the principles of the Declaration of Helsinki.

Participants aged 16–21 years with regular Instagram use for at least 6 months and active engagement with Reels content were included. All participants were required to stand independently for at least 60 s, have normal or corrected-to-normal vision, adequate cognitive capacity to follow instructions, and provide written informed consent. For participants under 18, written informed consent was obtained from parents/guardians and assent from participants. The selected age range (16–21 years) represents a developmental period characterized by ongoing maturation of sensory integration and postural control systems, alongside high levels of digital media engagement. Previous research has shown that postural control and sensory system maturation continue into late adolescence, supporting the relevance of this age range (de Sá et al., 2018). Including both late adolescents and young adults allowed the examination of postural responses within a behaviorally relevant and relatively homogeneous high-exposure group.

Exclusion criteria comprised a history of vestibular, neurological, or orthopedic disorders affecting balance, acute vertigo or upper respiratory infection within the previous 48–72 h, use of medications influencing postural control, alcohol intake within the preceding 24 h, uncorrected visual impairment, head trauma within the last 6 months, severe musculoskeletal pain, and diagnosed psychiatric disorders.

### Sample size calculation

Postural stability scores and descriptive statistics of the participants in the middle-to-late adolescent age group (*N* = 32) are presented in Table 1. Sample size was estimated *a priori* using G*Power version 3.1.9.4 (Faul et al., 2007) for a within-subject (repeated-measures) design. Parameter selection was guided by effect sizes reported in previous experimental studies investigating the acute effects of smartphone and social media use on postural control using comparable paradigms and sample characteristics. Considering the increased statistical efficiency of repeated-measures designs and prior evidence demonstrating detectable within-subject balance changes with similar or smaller cohorts, the planned sample size (*N* = 32) was deemed sufficient to detect moderate within-subject effects with adequate statistical power (*α* = 0.05, power = 0.90).

**Table 1:** Postural stability scores and descriptive statistics of participants in the middle-to-late adolescent age group included in the study sample.

Postural stability and functional balance parameters (CTSIB and LOS)	X	Sd	Min.	Max.
Composite	70.18	8.44	41.00	81.00
Somatosensory	95.62	12.85	31.00	100.00
Visual	83.93	13.24	49.00	100.00
Vestibular	88.93	9.62	60.00	100.00
Visual preference	96.25	4.65	80.00	100.00
LOS reaction time	0.89	0.43	0.31	2.20
LOS mean velocity	2.88	0.86	1.16	5.43
LOS excursion	53.02	15.24	29.50	95.00
LOS directional control	75.50	11.30	36.35	97.00
Descriptive variables				
Age	19.84	1.48	16.00	21.00
Height (cm)	165.8	6.2	156	178
Weight (kg)	60.7	8.7	50	75
BMI (kg/m^2^)	23.1	2.3	19.3	27.1

Category	Subgroups	Frequency (N)	Percentage (%)
Purpose of Instagram use	Social interaction – communication with friends	10	31.3
	Spending time – relieving boredom	7	21.9
	Following current events – staying informed	6	18.8
	Entertainment – sharing	8	25.0
	Increasing followers – self-promotion	1	3.1
Average daily time spent on Instagram during the past week	0–1 h	2	6.3
	1–2 h	12	37.5
	2–3 h	10	31.3
	3–4 h	4	12.5
	4–5 h	3	9.4
	5 h and more	1	3.1

CTSIB, Clinical Test of Sensory Interaction and Balance; LOS, Limits of Stability; X, mean; Sd, standard deviation; Min., minimum; Max., maximum; N, number of participants; %, percentage; h, hour(s).

### Data collection tools

Information regarding participants’ descriptive characteristics was collected using a Personal Information Form, while Instagram addiction levels were assessed using the Instagram Addiction Scale (IAS).

#### Personal information form

The Personal Information Form, developed by the researchers, included questions addressing participants’ descriptive characteristics, specifically age and Instagram usage habits. These habits comprised primary purpose of Instagram use and average daily time spent on Instagram during the previous week. Given that anthropometric characteristics may influence postural stability, basic demographic and anthropometric variables (sex, height, and weight) were also recorded, and these characteristics are presented in Table 1 in the Results section. Time spent on Instagram was obtained from the application’s built-in usage statistics. Participants were instructed to access this information via the in-app pathway (Profile → Settings → Your Activity → Time Spent) and report their daily average usage. This approach ensured objective measurement and minimized recall bias.

#### Instagram Addiction Scale (IAS)

The Instagram Addiction Scale was originally developed as the Test for Instagram Addiction (TIA) by D’Souza et al. (2018) to assess Instagram addiction levels in adolescents and adults. The Turkish adaptation, including validity and reliability analyses in a Turkish sample, was conducted by (Kavaklı and Inan, 2021).

The scale is a 5-point Likert-type instrument ranging from 1 (never) to 5 (always) and consists of 21 items across five subdimensions: lack of control, escapism, disengagement, obsession, and health and interpersonal problems. The Turkish version does not contain any reverse-coded items. The test–retest reliability coefficient of the scale was reported as 0.77 (Kavaklı and Inan, 2021). In the present study, internal consistency analysis was calculated and indicated excellent reliability, with a Cronbach’s alpha coefficient of 0.90 for the total scale.

#### Virtual reality based clinical test of sensory interaction on balance (CTSIB) and limits of stability (LOS) test

Participants were instructed to maintain an upright bipedal stance, barefoot, on a virtual reality–based static posturography system (Virtualis Balance VR, France), which integrates a force platform with immersive visual stimulation for high-resolution postural assessment. The system enables precise recording of center of pressure (CoP) displacement and sensory integration strategies under controlled visual and somatosensory conditions.

Postural stability was evaluated using the Clinical Test of Sensory Interaction on Balance adapted for virtual reality (CTSIB-VR). Participants stood with their arms relaxed at their sides and their feet positioned according to standardized foot placement guidelines. Two surface conditions were applied: a firm surface (direct contact with the force platform) and an unstable surface, achieved using a standardized foam pad (Airex Balance Pad Elite), to selectively perturb somatosensory input (Meldrum et al., 2012; Sherman et al., 2022).

Six sensory conditions were tested: (1) firm surface with eyes open, (2) firm surface with eyes closed, (3) firm surface with sway-referenced visual stimulation, (4) foam surface with eyes open, (5) foam surface with eyes closed, and (6) foam surface with sway-referenced visual stimulation (see Supplementary Table S1). Each condition lasted 20 s and was repeated three times. Brief rest periods were provided between trials. For statistical analysis, the mean of the three trials for each condition was used to improve measurement reliability and reduce trial-to-trial variability.

During the Reels viewing condition, participants performed only the eyes-open CTSIB conditions to allow continuous visual exposure to the stimulus. Eyes-closed conditions were assessed exclusively during the baseline measurement as part of the standard CTSIB protocol. These conditions were included to characterize general postural control and to ensure the absence of underlying balance impairment prior to experimental manipulation, rather than for direct comparison with the Reels viewing condition.

CTSIB sub-scores (somatosensory, visual, vestibular, and visual preference) were calculated using standard sensory analysis ratios derived from performance across specific test conditions, consistent with established Clinical Test of Sensory Interaction on Balance (CTSIB) and Sensory Organization Test (SOT) methodologies (Shumway-Cook and Horak, 1986; Khattar and Hathiram, 2012; Cohen et al., 2019).

In the VR-based posturography system, balance performance was quantified using system-derived stability metrics based on oscillatory body movements detected by the head-mounted display rather than relying solely on conventional force-platform–derived center-of-pressure (CoP) measurements (Virtualis, 2025). These oscillatory signals reflect postural sway in anterior–posterior and mediolateral directions, where greater amplitude and variability indicate reduced stability. The system internally processes and normalizes these oscillations to generate equilibrium scores ranging from 0 to 100, with higher scores indicating better postural control. This approach is conceptually consistent with established posturography models in which stability is derived from sway variability and its spatial characteristics (Le Perf et al., 2025). Equilibrium scores obtained for each condition were used as the primary performance metric for calculating CTSIB sub-scores, which were derived using standard ratio-based comparisons across sensory conditions.

Each sensory ratio was computed by comparing performance in a sensory-challenged condition to a baseline condition (eyes open, firm surface), following standard SOT-derived analytical frameworks. This ratio-based approach enables the isolation of sensory system contributions and reflects adaptive sensory reweighting mechanisms underlying postural control.

The somatosensory ratio was calculated as the ratio of performance in the eyes-closed firm surface condition to the eyes-open firm surface condition (Condition 2/Condition 1), reflecting reliance on proprioceptive input in the absence of vision. The visual ratio was derived from the eyes-open foam surface condition relative to eyes-open firm surface (Condition 4/Condition 1), indicating the contribution of visual input when somatosensory input is degraded. The vestibular ratio was calculated using performance in the eyes-closed foam surface condition relative to baseline (Condition 5/Condition 1), isolating vestibular contribution under conditions where both visual and somatosensory inputs are compromised. Visual preference was computed as the ratio of performance under visual conflict conditions to non-visual conditions (Condition3+Condition6)/(Condition2+Condition5) , reflecting the degree of reliance on inaccurate visual input (Nashner, 1993; Sung and Lee, 2025).

In all ratio calculations, the condition-specific values (e.g., Condition 2/Condition 1) correspond to the equilibrium scores generated by the VR-based CTSIB system for each condition. These equilibrium scores are derived from oscillatory body movements detected by the head-mounted display, reflecting postural sway variability (i.e., magnitude and dispersion of body oscillations over time) in anterior–posterior and mediolateral directions, and are normalized on a scale from 0 to 100, with higher values indicating better stability (Interacoustics, 2025). Therefore, CTSIB sub-scores in the present study were calculated using these processed equilibrium scores rather than raw center-of-pressure (CoP) signals.

These ratios provide a quantitative representation of multisensory integration processes in postural control. However, as highlighted in recent systematic evidence, substantial methodological heterogeneity exists across posturography studies in terms of task conditions, sway parameters, and calculation methods, underscoring the need for standardized reporting and interpretation of balance metrics (Julienne et al., 2024). Therefore, the present study adopts a transparent and explicitly defined computational framework to enhance reproducibility and comparability.

Although vestibular input was not directly manipulated, the CTSIB paradigm enables indirect assessment of vestibular contribution by systematically reducing or distorting visual and somatosensory inputs. In particular, the eyes-closed foam surface condition functionally isolates vestibular input by minimizing reliable visual and proprioceptive cues. Therefore, the vestibular sub-score is interpreted as reflecting the relative contribution of vestibular processing within a multisensory integration framework rather than a direct measure of vestibular function (Shumway-Cook and Horak, 1986; Cohen et al., 2019).

**Figure 1:**
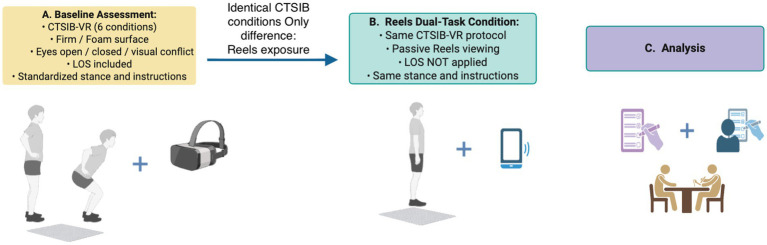
Schematic overview of the study design. Identical postural assessment conditions (CTSIB-VR protocol, stance, and instructions) were applied in both baseline and Reels conditions, with the only difference being passive Instagram Reels exposure during the second condition. Limits of Stability (LOS) testing was conducted only at baseline.

To simulate ecologically valid dual-task conditions, postural assessments were conducted both before and during Instagram Reels viewing (Figure 1). During the Reels condition, participants wore in-ear headphones and passively viewed algorithm-driven Instagram Reels content presented on a smartphone by the experimenter. Participants were instructed not to interact with the smartphone (e.g., *scrolling or touching the screen*) to minimize upper-limb movements and prevent task-induced postural artifacts.

The smartphone was held by the experimenter to minimize upper-limb movement artifacts that could influence center-of-pressure measurements and confound postural outcomes. By standardizing the viewing position, the effect of visual–cognitive load on postural stability could be isolated. Participants were assessed in a standing posture, as the primary aim of the study was to evaluate postural control, which cannot be reliably assessed in a seated position within VR-based posturography paradigms. Posturographic assessment relies on center-of-pressure dynamics obtained during upright stance. In addition, standing represents the fundamental biomechanical basis of locomotor activities (e.g., walking and directional changes) and provides a sensitive condition for detecting balance alterations. Prior evidence indicates that dual-task demands associated with smartphone use are most clearly reflected during upright stance (Nurwulan et al., 2015), whereas seated conditions may reduce postural demands and mask visually induced destabilizing effects.

The smartphone was positioned at a standardized viewing distance of approximately 40 cm, allowing consistent visual exposure while preserving the validity of postural measurements. The Reels content was drawn from participants’ own personal Instagram accounts, reflecting algorithm-driven, individualized content streams, in order to preserve ecological validity and naturalistic engagement. Participants were instructed not to interact with the content (e.g., scrolling or liking) to minimize movement-related artifacts.

During CTSIB-VR assessment, participants were instructed to stand upright in a standardized bipedal stance with their arms relaxed at their sides, maintaining a forward gaze and minimizing head and body movements. Foot position was kept consistent across trials in accordance with the device’s standardized placement guidelines. Each condition lasted 20 s and was repeated three times, with short rest intervals between trials to reduce fatigue effects. The 20-s trial duration was selected to capture acute concurrent postural responses while minimizing fatigue, attentional drift, boredom, and time-on-task effects that could confound interpretation under dual-task conditions.

Centre-of-pressure (CoP) data were acquired using the integrated force platform of the system at a fixed sampling rate, and postural stability parameters were automatically computed by the system software based on sway characteristics across conditions. The mean of three trials for each condition was used for statistical analysis to enhance measurement reliability. Functional balance capacity was additionally assessed using the Limits of Stabilit (LOS) test, which provides a quantitative evaluation of dynamic postural control by measuring the individual’s ability to voluntarily displace the center of gravity within the base of support. This paradigm allows characterization of anticipatory postural adjustments and dynamic balance strategies and has been used to assess functional balance across age groups and clinical populations (Faraldo-García et al., 2016). Participants were instructed to shift their center of gravity toward visually presented targets in eight directions (anterior, posterior, lateral, and diagonal) without lifting their feet. Reaction time, mean velocity, endpoint excursion, maximum excursion, and directional control parameters were recorded. Each trial was performed three times, and the mean of the three trials was used for statistical analysis to improve reliability and reduce trial-to-trial variability. The LOS test was applied only at baseline, as its execution requires active voluntary weight shifting; performing it during Reels viewing would introduce methodological inconsistency and substantial movement-related artifacts. The LOS test was included to characterize participants’ baseline dynamic balance capacity and to provide a reference measure of voluntary limits of postural control, complementing the sensory integration–focused CTSIB assessment. The LOS test was applied only at baseline, as its execution requires active voluntary weight shifting; performing it during Reels viewing would introduce methodological inconsistency and substantial movement-related artifacts. The LOS test was included only to characterize baseline functional balance capacity and voluntary postural control, complementing the sensory-organization focus of CTSIB rather than contributing directly to the primary pre–during Reels comparison. This distinction is consistent with the multidimensional nature of postural control, in which different balance tests capture different components of postural performance (Horak, 2006; Pollock et al., 2000).

Specifically, center of pressure (CoP) displacement was quantified in both the anteroposterior (AP) and mediolateral (ML) directions during each trial. From these signals, an equilibrium score was computed based on the magnitude of postural sway, reflecting CoP excursion relative to theoretical stability limits (Nashner, 1997; Guskiewicz, 2001). The equilibrium score was calculated using the following formulation:

Equilibrium score = [1 − (*θ* / θmax)] × 100

where θ represents the peak-to-peak CoP displacement converted into an equivalent sway angle in the anteroposterior plane, and θmax corresponds to the theoretical limit of stability (typically 12.5° in standard posturography paradigms). Higher scores indicate reduced sway and greater postural stability. These equilibrium scores were used as the primary CoP-derived performance metric and served as the basis for calculating CTSIB sensory ratios (somatosensory, visual, vestibular, and visual preference) through condition-specific comparisons.

### Statistical analysis

Prior to analysis, the dataset was screened for missing values, outliers, and data entry errors. Following these checks, the data were deemed suitable for statistical analysis. Normality of distribution was evaluated using skewness and kurtosis coefficients. The coefficients for the Instagram Addiction Scale scores and for the postural stability measures obtained during the first (pre-Reels viewing) and second (during-Reels viewing) assessments were within the range of −2.0 to +2.0, indicating that the assumption of normality was satisfied (George and Mallery, 2024). All statistical analyses were conducted using SPSS Statistics version 25 (IBM Corp., Armonk, NY, United States).

Differences in postural stability performance before and during Instagram Reels viewing were analyzed using the Paired Samples *t*-test, given the repeated-measures design and fulfillment of the normality assumption (Büyüköztürk, 2018). The association between change scores in postural stability parameters (Δ pre–during) and Instagram addiction levels was examined using Pearson’s product–moment correlation coefficient. To assess whether changes in postural stability varied according to Instagram usage characteristics (purpose of use and daily usage duration), the non-parametric Kruskal–Wallis *H* test was applied due to insufficient subgroup sample sizes. When significant differences were detected, *post hoc* pairwise comparisons were performed using the Mann–Whitney *U* test with Holm–Bonferroni adjustment to control for multiple comparisons. For correlation analyses, 95% confidence intervals were calculated using bias-corrected bootstrap resampling to improve estimation robustness. To control for Type I error inflation due to multiple comparisons, appropriate corrections (e.g., Holm–Bonferroni adjustment for *post hoc* tests) were applied where necessary.

## Results

Postural stability scores and descriptive statistics of the participants in the middle-to-late adolescent age group (*N* = 32) are presented in Table 1.

The primary outcome measure of this study was the CTSIB vestibular sub-score, reflecting vestibular-dependent postural stability under conditions of reduced visual and somatosensory input. Secondary analyses included baseline postural parameters, correlation analyses with Instagram addiction scores, and subgroup comparisons based on Instagram use characteristics.

Descriptive statistics for postural stability, functional balance, and Instagram use characteristics are summarized in Table 1. Composite score (*X* = 70.18, Sd = 8.44), Somatosensory score (*X* = 95.62, Sd = 12.85), Visual score (*X* = 83.93, Sd = 13.24), Vestibular score (*X* = 88.93, Sd = 9.62), Visual Preference score (*X* = 96.25, Sd = 4.65), LOS Reaction Time (*X* = 0.89, Sd = 0.43), LOS Mean Velocity (*X* = 2.88, Sd = 0.86), LOS Excursion (*X* = 53.02, Sd = 15.24), and LOS Directional Control (*X* = 75.50, Sd = 11.30). These CTSIB-derived scores represent normalized equilibrium-based ratios reflecting the relative contribution and reliability of somatosensory, visual, and vestibular inputs to postural control under different sensory conditions.

Participants’ ages ranged from 16 to 21 years (*X* = 19.84, Sd = 1.48). Regarding Instagram usage patterns, the most frequently reported primary purpose was social interaction and communication with friends (*N* = 10), followed by entertainment and content sharing (*N* = 8). In terms of average daily Instagram use during the preceding week, the highest proportion of participants reported spending 1–2 h per day (*N* = 12), followed by 2–3 h per day (*N* = 10).

**Figure 2:**
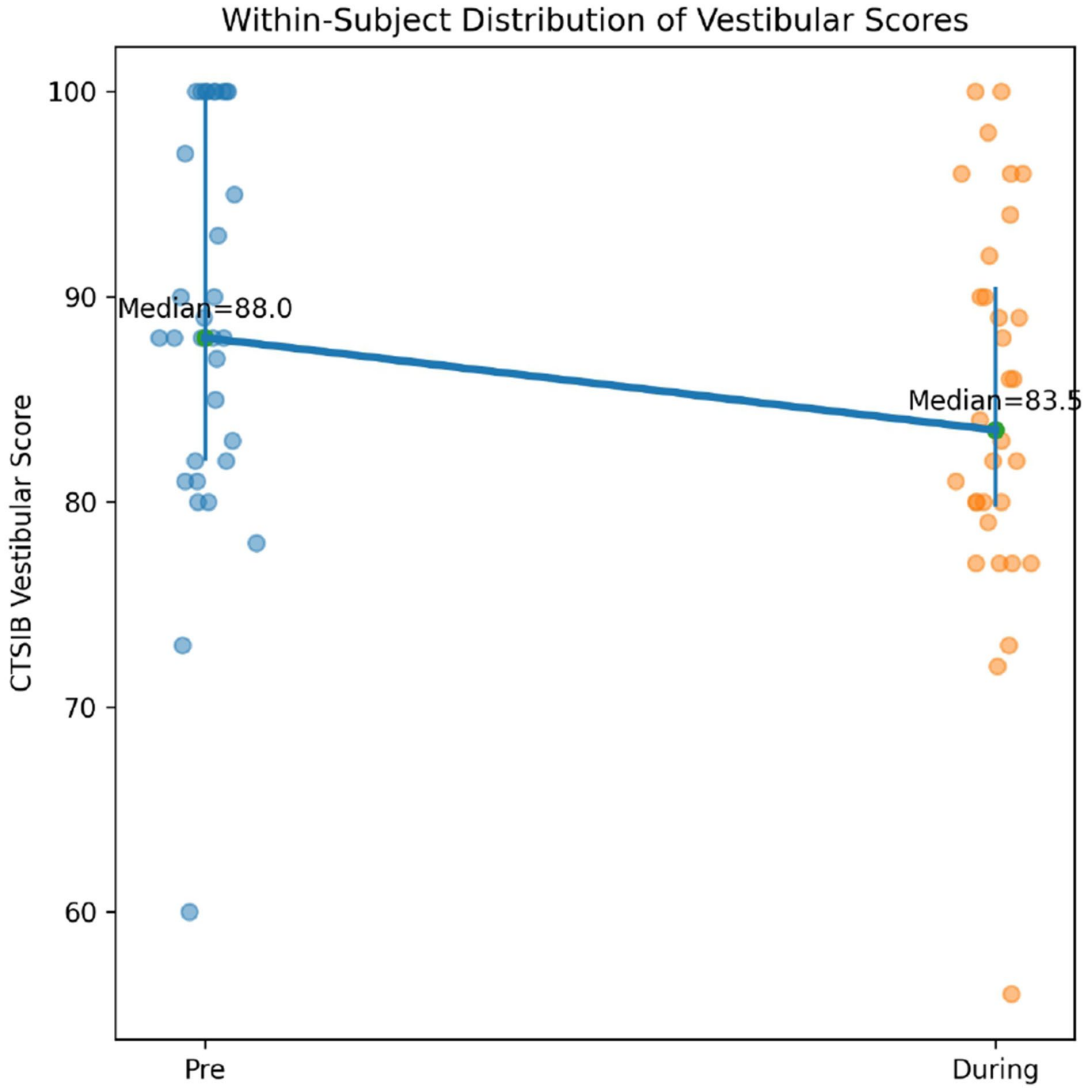
Within-subject distribution of CTSIB vestibular sub-scores before and during Instagram Reels viewing. Individual data points are shown for each participant. Median values are indicated with connecting lines, and vertical bars represent interquartile ranges. A reduction in vestibular scores during Reels viewing is observed.

Changes in vestibular-dependent postural stability were evaluated using the CTSIB vestibular sub-score. As shown in Table 2, the difference between CTSIB vestibular subscores measured before Instagram Reels viewing and those obtained during viewing was statistically significant [*t*(31) = 2.161, *p* = 0.038]. Specifically, vestibular stability scores during Reels viewing (*X̄* = 84.68 ± 9.48) were significantly lower than pre-viewing scores (*X̄* = 88.93 ± 9.62). This finding indicates that Instagram Reels consumption as a form of digital content exposure acutely reduced vestibular-related postural stability, thereby impairing vestibular-related postural control in adolescents. As illustrated in Figure 2, vestibular scores decreased at the group level, while individual variability in responses was also observed.

**Table 2:** Paired-samples *t*-test comparison of CTSIB vestibular subscore scores before and during Instagram Reels viewing.

Postural stability	Measurement	*N*	Mean (X)	SD	df	*t*	*p*
CTSIB-vestibular	Pre-viewing	32	88.93	9.62	31	2.161	0.038*
	During viewing	32	84.68	9.48			

CTSIB-VR, Clinical Test of Sensory Integration of Balance–Virtual Reality; values are presented as mean ± SD; *p* < 0.05: statistically significant (paired-samples *t*-test). *p < 0.05 indicates statistical significance.

The relationship between vestibular stability change and behavioral measures was assessed using correlation analysis. As shown in Table 3, the association between the change in vestibular stability (*Δ* = Pre − During) and Instagram addiction scores was examined using Pearson’s correlation analysis. The results revealed a moderate, statistically significant negative correlation between the vestibular score difference and Instagram addiction scores (*r* = −0.353, *p* = 0.048), with a 95% confidence interval that did not cross zero (95% CI [−0.62, −0.01]), indicating a reliable inverse association. Higher Instagram addiction scores were associated with smaller vestibular stability change scores (*r* = −0.353, *p* = 0.048).

**Table 3:** Correlation between the change in vestibular scores (Pre–During Reels viewing) and Instagram addiction levels.

Variables	Statistics	Δ vestibular score (Pre–During)	Instagram addiction score
Δ Vestibular score (Pre–During)	Correlation Coefficient	1	−0.353*
	Sig. (2-tailed)		0.048
	N	32	32
Instagram addiction score	Correlation Coefficient	−0.353*	1
	Sig. (2-tailed)	0.048	
	N	32	32

Δ: difference score (Pre − During); values are Pearson correlation coefficients; *p* < 0.05: statistically significant. *p < 0.05 indicates statistical significance.

**Figure 3:**
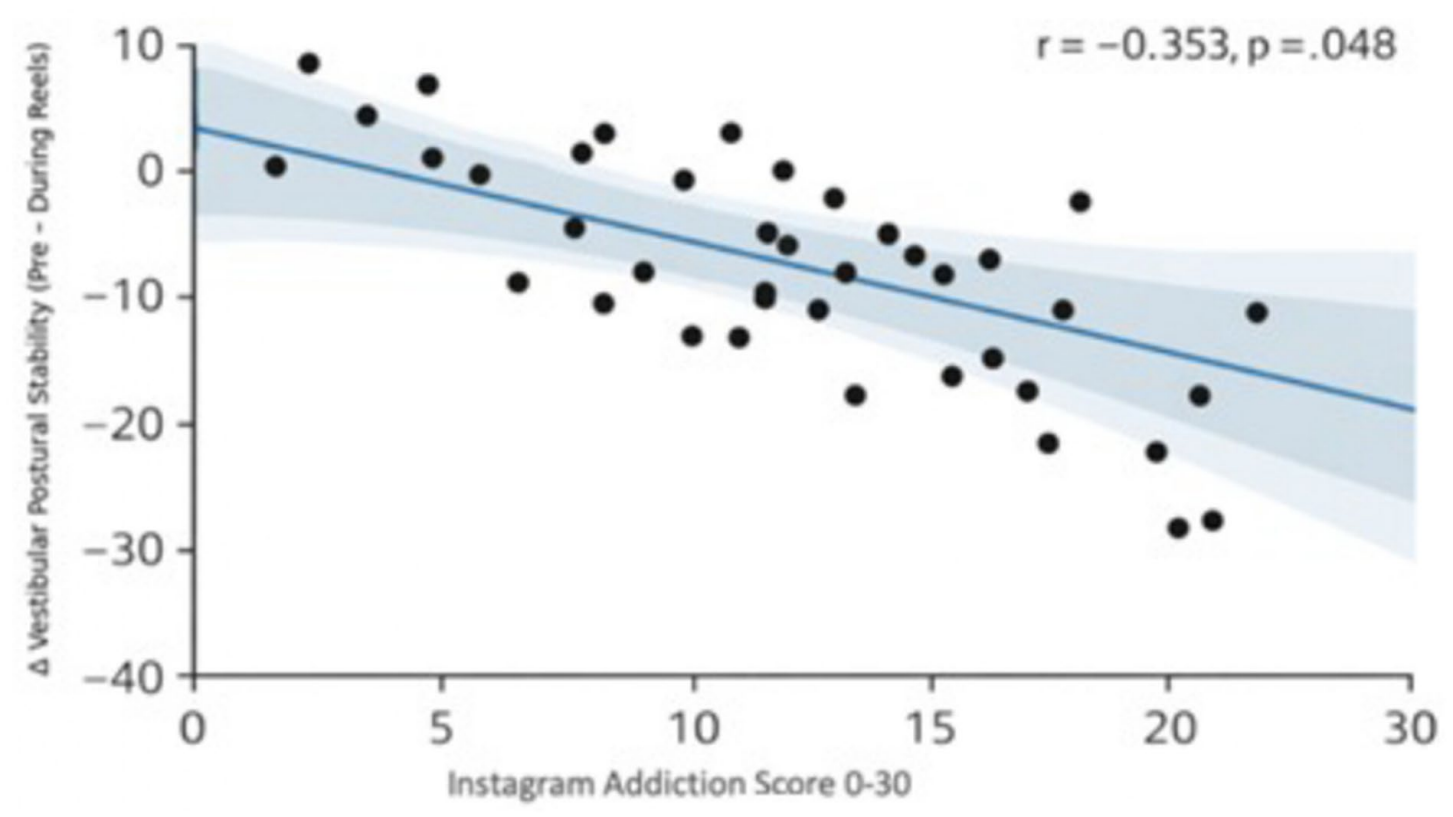
Scatter plot showing the association between Instagram addiction scores and the change in CTSIB vestibular sub-score calculated as pre-Reels minus during-Reels values (Δ = Pre – During).

Change scores were calculated as *Δ* = Pre − During; therefore, smaller Δ values represent a smaller reduction in vestibular stability during Reels viewing, consistent with a pattern of partial adaptation or compensatory postural control (see Figure 3).

Subgroup analyses were conducted to examine whether vestibular stability changes differed according to Instagram use characteristics. As shown in Table 4, the differences between CTSIB vestibular subscore obtained before and during Instagram Reels viewing were compared across different Instagram use purposes using the Kruskal–Wallis *H* test. The analysis revealed no statistically significant differences among the groups [*χ*^2^(4, *n* = 32) = 1.277, *p* = 0.865]. No significant differences were observed across Instagram use purpose categories [*χ*^2^(4, *n* = 32) = 1.277, *p* = 0.865].

**Table 4:** Comparison of vestibular change scores across Instagram use purposes.

Measure	Purpose of Instagram use	*N*	Mean Rank	df	*X* ^2^	*P*	Difference
Δ Vestibular score (Pre–During)	(1) Social interaction/communication	10	16.35	4	1.277	0.865	–
	(2) Passing time/boredom relief	7	15.79				
	(3) Following news/staying informed	6	15.17				
	(4) Entertainment/sharing	8	19.13				
	(5) Increasing followers/promotion	1	10.00				
	Total	32					

Δ: difference score (Pre − During); *χ*^2^: Kruskal–Wallis *H* statistic; *p* < 0.05: statistically significant.

**Figure 4:**
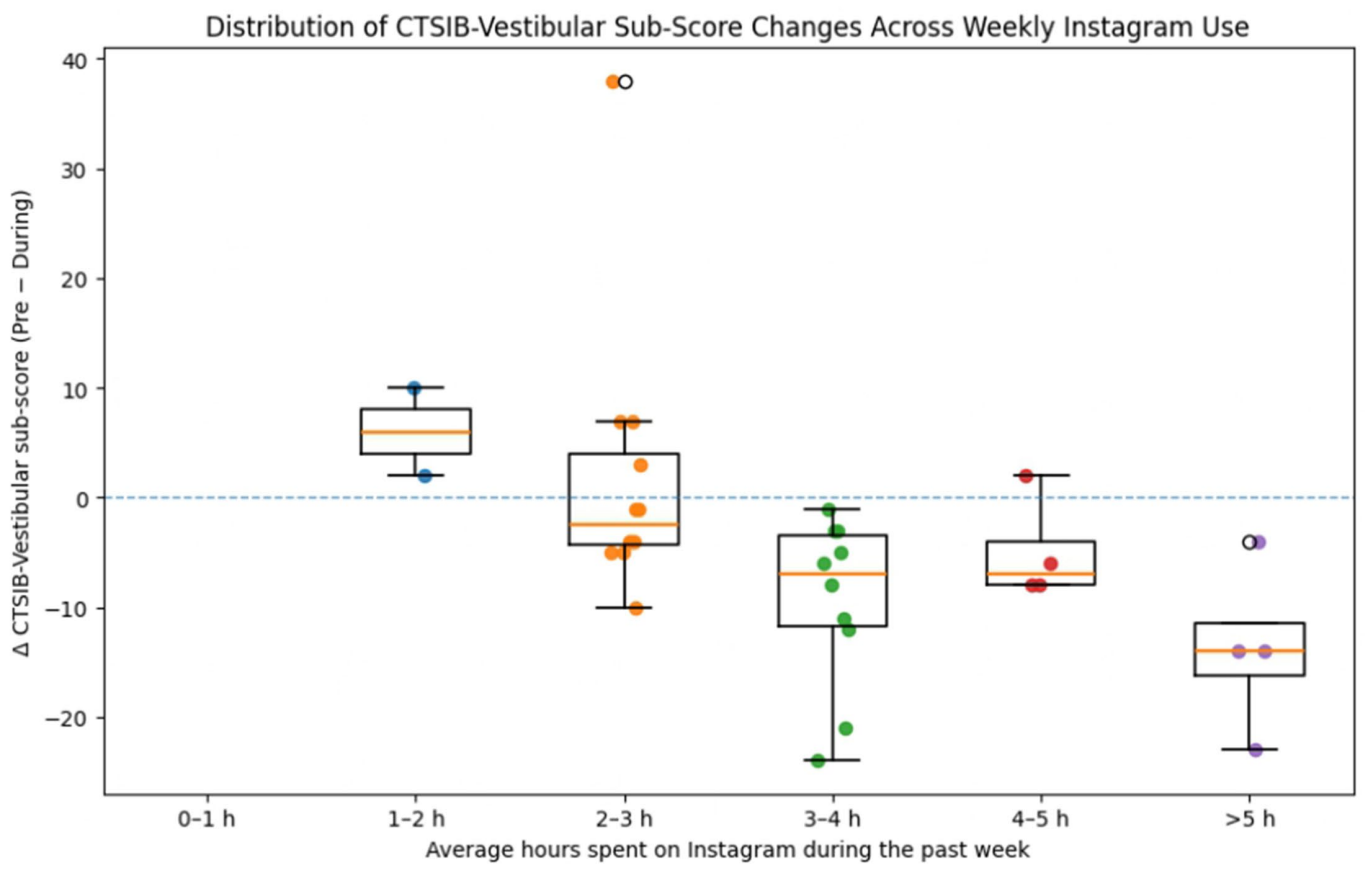
Distribution of changes in CTSIB-Vestibular sub-scores (Δ = Pre – During) across categories of average hours spent on Instagram during the past week. Negative values indicate greater reductions in CTSIB-Vestibular sub-scores during Reels viewing. Boxplots represent median and interquartile ranges, and individual data points are shown for each participant. The figure is intended to illustrate the distribution and variability of individual responses across usage categories; given the relatively small sample size in some subgroups and the substantial within-group variability, these visual patterns should be interpreted cautiously.

Subgroup analyses were conducted to examine whether vestibular stability changes differed according to Instagram use characteristics. As presented in Table 5, CTSIB vestibular subscore (Δ = Pre − During) differed significantly across categories of daily Instagram use duration [*χ*^2^(5, *n* = 32) = 13.375, *p* = 0.020]. *Post hoc* pairwise comparisons using the Mann–Whitney *U* test revealed that participants who spent 1–2 h per day on Instagram exhibited significantly smaller change scores than those who used Instagram 0–1 h per day. Likewise, individuals in the 2–3 h and 4–5 h groups showed significantly smaller differences compared with the 1–2 h group. As illustrated in Figure 4, CTSIB-vestibular sub-score changes (Δ = Pre − During) are distributed across categories of average weekly Instagram use, with a tendency toward smaller reductions in participants with higher exposure levels. However, given the substantial within-group variability and the relatively small sample sizes in some categories, these patterns should be interpreted with caution.

**Table 5:** Comparison of vestibular change scores across daily Instagram use duration.

Measure	Daily Instagram use (past week)	*N*	Mean rank	df	*X* ^2^	*P*	Difference
Δ Vestibular score (Pre–During)	(1) 0–1 h	2	28.75	5	13.375	0.020*	1–2
	(2) 1–2 h	12	21.71				2–3
	(3) 2–3 h	10	12.15				2–5
	(4) 3–4 h	4	14.75				
	(5) 4–5 h	3	8.33				
	(6) > 5 h	1	4.50				
	Total	32					

Δ: difference score (Pre − During); *χ*^2^: Kruskal–Wallis *H* statistic; *p* < 0.05: statistically significant; pairwise comparisons performed using Mann–Whitney *U* test. *p < 0.05 indicates statistical significance.

Overall, these findings indicate that as daily Instagram exposure increases, the magnitude of Reels-related vestibular destabilization progressively decreases. Vestibular stability change scores differed significantly across daily Instagram use duration categories [*χ*^2^(5, *n* = 32) = 13.375, *p* = 0.020].

## Discussion

This study investigated whether passive exposure to algorithm-driven Instagram Reels, characterized by high visual flow and continuous attentional engagement, acutely alters postural stability in healthy adolescents and young adults (16–21 years), and whether the magnitude of this effect is modulated by Instagram addiction severity and habitual usage patterns. Three principal findings emerged. First, vestibular-related postural stability significantly decreased during Reels viewing compared with baseline, indicating an acute destabilizing effect of short-video exposure. Second, higher Instagram addiction scores were associated with smaller pre–during differences, suggesting reduced acute susceptibility among individuals with greater habitual exposure. Third, daily Instagram usage duration similarly moderated this effect, with heavier users exhibiting smaller destabilization, whereas the purpose of Instagram use showed no meaningful association. Collectively, these findings are consistent with a dual pattern: acute digital visual load compromises postural control, while chronic exposure appears compatible with partial behavioral or sensory adaptation. Importantly, this effect was specific to the vestibular-related sub-score, as no significant changes were observed in other CTSIB components, indicating that the observed destabilization does not reflect a global impairment of postural stability. The present findings should be interpreted within the framework of sensory reweighting rather than direct vestibular activation. Importantly, given the absence of a dedicated visual or non-social-media control condition, these effects cannot be attributed specifically to Instagram Reels per se and may instead reflect more general effects of visual–cognitive load or dual-task demands on postural control. The CTSIB does not manipulate vestibular input per se but creates conditions in which vestibular information becomes relatively more critical for postural control. In this context, foam versus firm surface conditions play a critical role by systematically reducing somatosensory reliability, thereby shifting sensory weighting toward vestibular and visual inputs; these manipulations are inherently embedded within CTSIB-derived sub-scores rather than analyzed as separate experimental factors.

### Acute Reels exposure and vestibular postural destabilization

The observed reduction in vestibular-related postural stability during Reels viewing aligns with prior findings demonstrating that smartphone use increases postural sway and impairs balance under visually demanding conditions (Onofrei et al., 2020; Noll et al., 2024; Lee and Son, 2025). Studies in healthy young populations have consistently shown that digital activities such as talking, texting, note-taking, selfie capturing, and social media posting increase postural sway and reduce stability margins, even during quiet standing (Noll et al., 2024). In line with this evidence, Onofrei et al. (2020) reported significantly greater center-of-pressure path length, confidence area, and maximal CoP speed during smartphone talking and texting compared with a no-phone condition. Although our paradigm involved passive viewing rather than active interaction, the convergence in effect direction suggests that visually and cognitively demanding screen exposure alone is sufficient to degrade postural control. Notably, experimental data further indicate that even brief smartphone exposure—on the order of 10 min—is associated with reduced stability scores and increased center-of-pressure path length in adolescents, affecting both static and dynamic balance performance (Anter and Al-Tohamy, 2024).

Mechanistically, this destabilization likely reflects the combined influence of increased cognitive load and altered sensory weighting. Short-form video platforms such as TikTok and Instagram Reels are designed to maximize attentional capture through rapid visual transitions and algorithm-driven content delivery, resulting in sustained cognitive engagement even during passive viewing (Montag et al., 2021). According to dual-task and attentional resource theories, postural control is not entirely automatic but requires a finite share of central processing capacity; when cognitive load increases, fewer resources remain available for postural regulation, leading to increased sway and reduced stability (Woollacott and Shumway-Cook, 2002; Huxhold et al., 2006). Converging neurocognitive evidence suggests that short-form video exposure may further vestibular- related postüral regulation by affecting attentional control processes. Luo et al. (2025) reported that exposure to short-form videos is associated with reduced cognitive control capacity under visually demanding conditions. This reduction in cognitive control may limit the efficient allocation of attentional resources required to maintain stable posture, thereby contributing to vestibular-related instability observed during Reels viewing.

In parallel, the continuous and dynamic nature of Reels content may introduce visual perturbations that challenge postural control, consistent with studies showing that visually induced perturbations increase gait variability and reduce stability under dynamic visual conditions (Wilson et al., 2025). Experimental studies manipulating smartphone exposure duration in adolescents have shown that prolonged screen engagement exacerbates postural instability particularly under sensory-challenging conditions, consistent with visual fatigue and sensory conflict mechanisms (Bernard-Arevalo et al., 2023). Although our cohort consisted of healthy individuals and the primary change was detected in vestibular-related parameters, the conceptual framework remains consistent: sustained, visually dominant stimulation can disrupt multisensory integration processes that are critical for maintaining vestibular-dependent postural stability.

Importantly, vestibular and somatosensory inputs were not directly manipulated in this paradigm. Instead, the continuous and high-salience visual stimulation likely induced a relative shift in sensory weighting by increasing visual dominance and attentional capture, thereby functionally reducing the contribution of non-visual sensory inputs during postural control. This interpretation is consistent with sensory reweighting models demonstrating that postural stability can be altered through relative changes in sensory reliance even in the absence of direct perturbation of all sensory channels (Liu et al., 2024; Li et al., 2025).

Notably, the acute effect in our data was most evident in the CTSIB-VR vestibular-related stability score, which supports a sensory-weighting interpretation but also cautions against overgeneralizing the finding to global postural control. Consistent with a sensory-conflict account, recent VR gait work shows that visually applied perturbations—particularly in the medio-lateral axis—reduce locomotor stability and increase gait variability, with stronger destabilizing effects in head-mounted displays, underscoring the capacity of high-salience visual motion to challenge balance control (Wilson et al., 2025).

### Moderation by addiction severity and daily use

A novel and theoretically meaningful contribution of this study lies in the moderation pattern observed across addiction severity and daily usage duration. The negative correlation between Instagram addiction scores and the magnitude of postural destabilization indicates that individuals with higher habitual exposure exhibit reduced acute sensitivity to short-video viewing. Similarly, greater daily Instagram use was associated with smaller pre–during differences, supporting the interpretation of partial adaptation or tolerance-like processes. However, this association should be interpreted with caution, as visual inspection of the data distribution suggests that the effect may be partially influenced by extreme values. Therefore, these findings should not be considered conclusive, and further studies with larger samples are needed to confirm the robustness of this relationship.

This adaptation signal is biologically plausible. Repeated exposure to complex visual environments can induce recalibration within multisensory integration networks, a mechanism widely exploited in virtual reality–based rehabilitation to improve balance control, even in neurological populations (Ozkul et al., 2020). Although our study is cross-sectional, the observed pattern is compatible with central sensory reweighting, whereby sustained visual dominance gradually reshapes vestibular and somatosensory contributions to postural regulation.

A complementary interpretation involves behavioral compensation. Frequent users may develop implicit strategies—such as subtle stance stiffening, micro-adjustments in pressure distribution, or optimized gaze control—that attenuate destabilization during visually demanding tasks. Consistent with dual-task gait paradigms, individuals often preserve cognitively salient task performance by modifying motor strategies (Strubhar et al., 2015). In our design, which restricted upper-limb movement, adaptation likely reflects improved efficiency in processing high-rate visual information, thereby reducing attentional capture and preserving stability, consistent with evidence showing that short-form video use negatively impacts executive attentional control (Yan et al., 2024).

Importantly, this moderation pattern should not be interpreted as direct evidence of beneficial adaptation, but rather as reflecting central recalibration and compensatory control strategies that mitigate acute perturbation without necessarily enhancing overall postural safety in unpredictable real-world contexts.

Most prior studies have emphasized the acute destabilizing effects of smartphone exposure during interactive tasks such as talking or note-taking (Özdemir et al., 2024), texting (Strubhar et al., 2015; Gulatowska and Błażkiewicz, 2025), selfie taking and social media posting (Noll et al., 2024), or prolonged exposure durations (Jouira et al., 2023). These paradigms typically model smartphone use as a uniform perturbation, without explicitly considering habitual exposure as a moderating factor. In contrast, our findings add a novel layer by demonstrating that, although Reels viewing acutely disrupts balance, the magnitude of this disruption is systematically modulated by addiction severity and daily usage.

This divergence can be explained by two methodological features. First, previous paradigms relied largely on active, motorically demanding tasks that constrain upper-limb movement and amplify both cognitive and biomechanical postural load (Noll et al., 2024; Özdemir et al., 2024; Gulatowska and Błażkiewicz, 2025). Our paradigm isolated passive high-visual-flow exposure, a condition under which habituated users may deploy more automatized perceptual and postural strategies, thereby reducing acute destabilization. Second, while duration-based manipulations accentuate cumulative visual fatigue and sensory conflict (Jouira et al., 2023), our moderation analysis highlights how exposure history may reshape sensory weighting and compensatory control, conceptually aligning with sensory recalibration principles described in rehabilitation contexts (Ozkul et al., 2020). Thus, rather than contradicting the literature, our findings extend it by showing that acute digital perturbations are not uniform but are systematically modulated by habitual exposure.

The absence of a significant effect of Instagram usage purpose suggests that the destabilizing influence of Reels viewing is driven predominantly by sensory and cognitive exposure characteristics rather than by motivational or contextual factors. This null result supports a parsimonious mechanistic model in which postural disruption is governed mainly by visual flow intensity, attentional capture, and habitual exposure, rather than by subjective reasons for platform engagement. Given the limited subgroup sizes, this result should be interpreted conservatively, yet it strengthens the argument that the observed effects are primarily neurocognitive rather than psychosocial in nature.

### Ecological validity and real-world implications

Most existing smartphone–balance studies have relied on controlled laboratory-based tasks such as texting, talking, or simple interactive smartphone paradigms, which, although experimentally robust, only partially reflect the complexity and continuity of real-world digital behaviors (Lamberg and Muratori, 2012; Schabrun et al., 2014; Strubhar et al., 2015). By specifically targeting passive short-form video consumption, characterized by sustained attentional capture and high visual flow, the present study provides a partially ecologically valid model of digital exposure by capturing passive short-form video viewing; however, it does not fully reflect real-world interaction patterns such as active scrolling and user engagement. Therefore, the ecological validity of the present paradigm should be interpreted with caution (Montag et al., 2021).

Our findings indicate that even quiet-standing postural control is measurably compromised during Instagram Reels viewing. This observation is consistent with prior evidence demonstrating that concurrent smartphone use degrades postural and gait stability, increases center-of-pressure displacement, and reduces dynamic stability margins during standing and walking tasks (Schabrun et al., 2014; Strubhar et al., 2015; Noll et al., 2024). Translating these laboratory observations into daily contexts, such digital-induced destabilization may plausibly elevate fall risk or balance loss during activities that demand stable posture under dynamic environmental conditions (e.g., obstacle negotiation and pedestrian tasks under texting-related distraction) (Licence et al., 2015).

Prior work has consistently shown that dual-task demands exacerbate postural sway in older adults, a population already characterized by age-related declines in muscle strength, sensory integration efficiency, and balance control (Bergamin et al., 2014). In this context, examining the effects of visually and cognitively demanding digital content in a younger, neurologically healthy population provides a critical reference framework. By isolating acute digital visual load effects in an age group with relatively intact vestibular and musculoskeletal function, the present study establishes a baseline against which future investigations in older adults and clinical populations can be more accurately interpreted. Specifically, this baseline reflects normative postural responses to short-form digital visual exposure in healthy adolescents and young adults with fully developed vestibular systems, providing a reference framework for comparison with populations exhibiting vestibular disorders, neurological conditions, or age-related balance impairments (Micarelli et al., 2020).

Although healthy adolescents and young adults may compensate effectively through rapid postural adjustments and attentional reallocation, the same visual–cognitive perturbation may carry substantially greater clinical relevance for individuals with vestibular disorders, neurological disease, or anxiety-spectrum conditions, in whom sensory reweighting capacity and attentional reserve are already compromised (Meldrum et al., 2012; Staab et al., 2017). In this context, the present findings provide a mechanistic bridge between everyday digital behaviors and balance control, supporting the need to consider short-form video exposure as a potentially meaningful modifier of postural stability in both healthy and clinical populations.

Several limitations should be acknowledged. First, although repeated-measures designs enhance statistical efficiency, the modest sample size constrains precision, particularly for subgroup analyses, and limits generalizability. Second, restricting manual interaction minimized movement artifacts but reduced ecological realism, as active scrolling and touch-based engagement represent core features of real-life Reels consumption. Finally, the observed moderation patterns cannot disentangle true sensory adaptation from stable individual differences, underscoring the need for longitudinal designs.

Third, the absence of an additional control condition (e.g., static image viewing or a non-social-media cognitive task) limits the ability to fully disentangle general visual-cognitive load from Reels-specific effects. However, the study was intentionally designed to evaluate the acute postural impact of ecologically valid Instagram Reels exposure. Given that Reels viewing constitutes a composite multisensory stimulus involving dynamic visual flow, sound, attentional capture, and individualized content engagement, stricter standardization may have reduced ecological validity while introducing additional interpretive constraints.

Finally, the absence of a post-viewing assessment precludes determination of whether the observed effects reflect transient responses, order effects, or time-on-task influences. Additionally, the observed moderation patterns cannot fully disentangle true sensory adaptation from stable individual differences, underscoring the need for longitudinal designs.

Future research should adopt longitudinal designs to directly test whether repeated short-video exposure induces progressive sensory recalibration or behavioral compensation. Integrating objective markers of visual fatigue, attentional load, and gaze behavior may clarify mechanistic pathways. Expanding paradigms to include dynamic balance tasks and high-risk clinical populations will further enhance translational relevance and define clinical thresholds for digital-exposure-related balance impairment.

## Conclusion

Passive exposure to Instagram Reels is associated with an acute reduction in vestibular-related postural stability in healthy adolescents and young adults, consistent with a digital cognitive–sensory load effect. Higher addiction severity and greater daily usage are associated with attenuated destabilization, yielding a pattern compatible with partial adaptation or compensatory control. These findings provide preliminary evidence that short-form video consumption can measurably alter postural regulation while highlighting the complex interplay between acute perturbation and habitual exposure.

## Data Availability

The raw data supporting the conclusions of this article will be made available by the authors, without undue reservation.
