# Supplementary figures and images for: Acute changes in postural stability during Instagram Reels viewing using virtual reality–based posturography
Source: Front Behav Neurosci. 2026 Apr 29;20:1808641. doi: 10.3389/fnbeh.2026.1808641 (PMC13168145; doi:10.3389/fnbeh.2026.1808641)

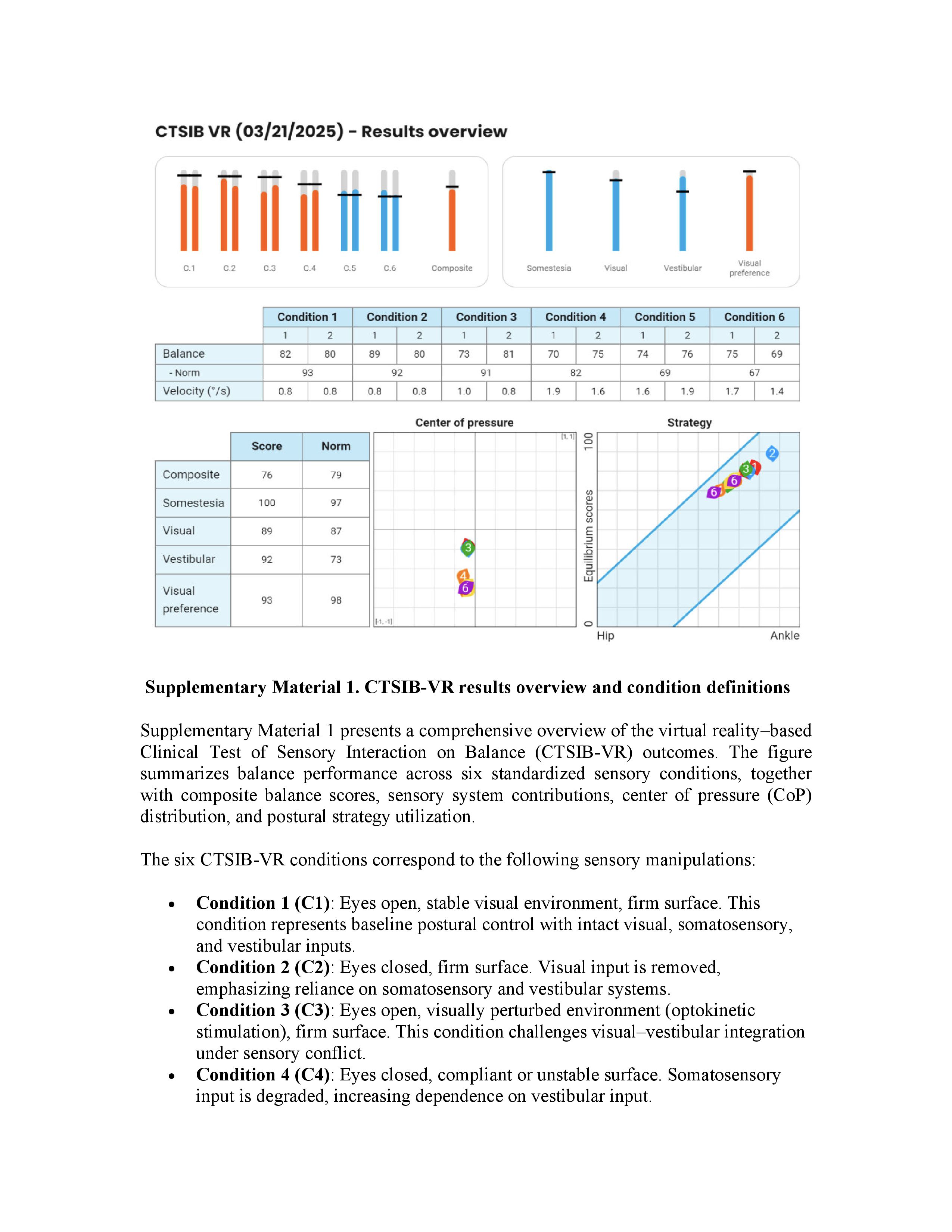

Supplement: Supplementary file 1 [file Data_Sheet_1.ZIP › sup. 1_1.jpg]

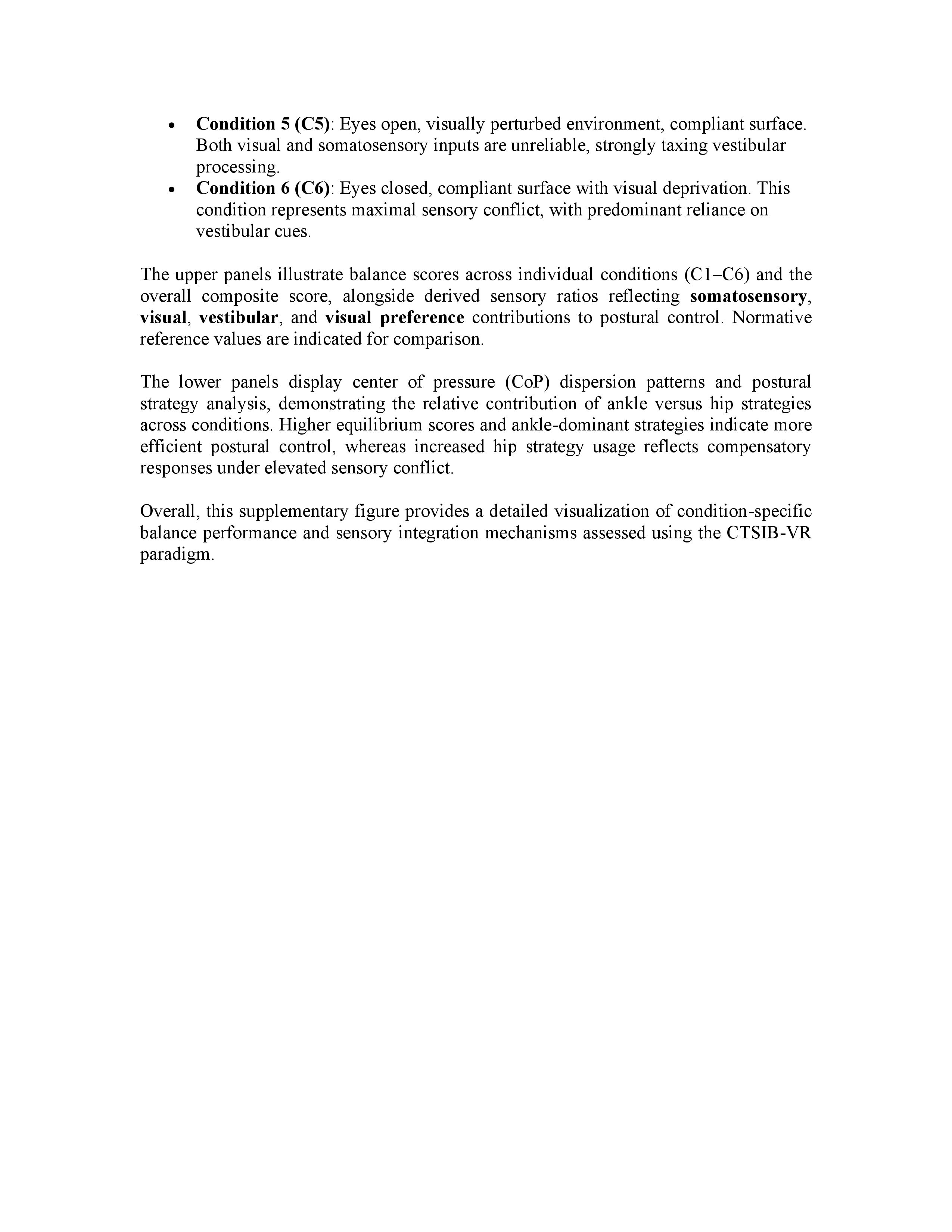

Supplement: Supplementary file 1 [file Data_Sheet_1.ZIP › sup. 1_2.jpg]
